# The Victoria West: earliest prepared core technology in the Acheulean at Canteen Kopje and implications for the cognitive evolution of early hominids

**DOI:** 10.1098/rsos.170288

**Published:** 2017-06-28

**Authors:** Hao Li, Kathleen Kuman, Matt G. Lotter, George M. Leader, Ryan J. Gibbon

**Affiliations:** 1 Key Laboratory of Vertebrate Evolution and Human Origins, Institute of Vertebrate Paleontology and Paleoanthropology, Chinese Academy of Sciences, Beijing 100044, People's Republic of China; 2 School of Geography, Archaeology and Environmental Studies, University of the Witwatersrand, Johannesburg WITS 2050, South Africa; 3 Department of Anthropology and Archaeology, University of Pretoria, Lynnwood Road, Pretoria 0083, South Africa; 4 Department of Anthropology, University of Pennsylvania, 3260 South Street, Philadelphia, PA, USA; 5 Department of Geological Sciences, University of Cape Town, Rondebosch 7701, South Africa

**Keywords:** Victoria West, Levallois, prepared core technology, Early Acheulean, cognitive evolution

## Abstract

Prepared core technology illustrates in-depth planning and the presence of a mental template during the core reduction process. This technology is, therefore, a significant indicator in studying the evolution of abstract thought and the cognitive abilities of hominids. Here, we report on Victoria West cores excavated from the Canteen Kopje site in central South Africa, with a preliminary age estimate of approximately 1 Ma (million years ago) for these cores. Technological analysis shows that the Victoria West cores bear similarities to the ‘Volumetric Concept’ as defined for the Levallois, a popular and widely distributed prepared core technology from at least 200 ka (thousand years ago). Although these similarities are present, several notable differences also occur that make the Victoria West a unique and distinctive prepared core technology; these are: elongated and convergent core shapes, consistent blow directions for flake removal, a predominance of large side-struck flakes, and the use of these flakes to make Acheulean large cutting tools. This innovative core reduction strategy at Canteen Kopje extends the roots of prepared core technology to the latter part of the Early Acheulean and clearly demonstrates an increase in the cognitive abilities and complexities of hominids in this time period.

## 1. Introduction

The Acheulean (approx. 1.7–0.3 Ma) has long been regarded as a highly successful, stable technological adaptation [1–5]. This stability is in part demonstrated by the continuity of tool types, including handaxes, picks and cleavers (collectively referred to here as large cutting tools—LCTs), which persist in Africa from their first occurrence at approximately 1.76 Ma until the shift to the Middle Stone Age (MSA) at approximately 200 ka [6–9]. In the Eurasian continent, the Acheulean techno-complex occurs earliest at the Attirampakkam site in India at approximately 1.5 Ma and in the Ubeidiya site in Israel at approximately 1.4 Ma, and is gradually replaced by the Middle Palaeolithic techno-complex at around 200 ka [10–12]. The mismatch between this long time span and the lack of technological innovations in the Acheulean was referred to as part of Isaac's ‘great puzzle’ [13,14].

However, increasing evidence in recent years is changing this understanding and some considerable advances in technological behaviour of Acheulean hominids have been detected. For instance, the appearance of blade technology in the Acheulean is now securely dated to approximately 0.5 Ma in the Kapthurin Formation of Kenya [15], and to a similar period at the South African site of Kathu Pan 1 [16]. The earliest hafting, which requires multi-tasking and an understanding of the composite tool concept [17], has also been suggested for points at Kathu Pan 1 [18]. In addition to these developments in lithic technology, the preservation of organic remains also shows innovations in the Acheulean, the most exciting being the controlled use of fire. Burned seeds and wood at the site of Gesher Benot Ya'aqov (GBY) in Israel are good indicators for the use of fire at around 0.7 Ma [19]. More recently, a microstratigraphic study of Wonderwerk Cave in South Africa demonstrates that the ability to control fire appeared even earlier, at approximately 1.0 Ma, with evidence for burned bone and ashed plant remains [20]. Therefore, it is clear that during the Acheulean we see several important changes in technological development, all of which contribute significantly to improving our understanding of hominid behavioural and cognitive evolution; however, most of these innovations occur during the Middle and Later Acheulean (1.0–0.3 Ma; [8]).

Cores in the Palaeolithic age of human prehistory refer to the knapped stones that were used to produce flakes with sharp edges. In this paper we present a detailed technological study of Victoria West cores, which are specially prepared to produce a single large flake thought to be used as blanks for handaxes and cleavers. These cores were first recognized almost a century ago in South Africa and were suggested to have a relationship with Levallois technology in the MSA [21–27], the most developed form of core preparation. However, our full understanding of the meaning and significance of Victoria West core technology has been limited by our inability to date and resolve the chronology of these cores. The Victoria West core sample presented here was systematically excavated from Canteen Kopje (Kopje means small hill) in South Africa. This assemblage has an age estimate of 0.8–1.1 Ma and is described in Beaumont and Vogel [28] and Leader [29].

## 2. Material and methods

### 2.1. Canteen Kopje

**Figure 1.**
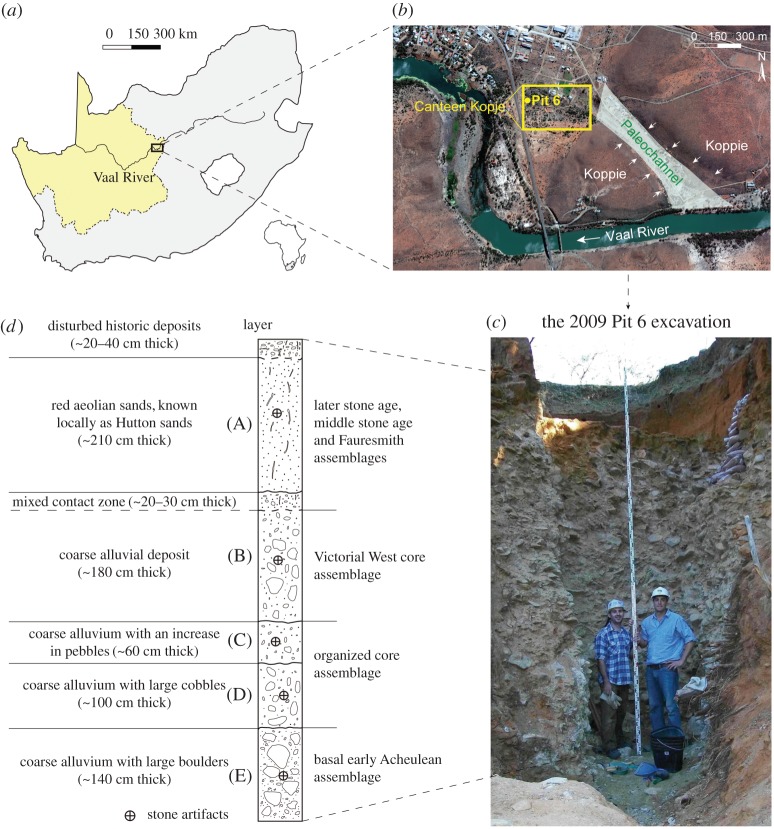
Location of Canteen Kopje in the Northern Cape Province (boundary shown in light yellow) of South Africa (*a*,*b*) and the stratigraphy of the Pit 6 excavation (*c*,*d*).

Canteen Kopje is an open-air alluvial site located along the Vaal River, in the Northern Cape Province of South Africa (figure 1*a*,*b*). The site is about 130 km southeast of the famous Wonderwerk Cave, within which early evidence for the controlled use of fire is found in an Acheulean layer [20], and is about 30 km northwest of Kimberley. Due to extensive historic mining work for diamonds, abundant stone artefacts were retrieved from the site in thick alluvial deposits of the ancient Vaal River. Archaeological excavations carried out at Canteen Kopje in 1997 confirmed the presence of Victoria West cores in the uppermost alluvial deposit at the site [30,31]. Along the Vaal River, other Acheulean sites containing the Victoria West cores have also been discovered, such as Pniel 1 and Pniel 6, along with a wider distribution of surface collected material in the interior of South Africa [32].

The Victoria West core sample studied here comes from the Pit 6 excavation, conducted in 2009 and led by G. M. Leader [29], R. J. Gibbon and K. Kuman (figure 1*b*). In total, more than 15 000 artefacts were excavated from Pit 6, and three distinct technological assemblages have been identified. These include a basal early Acheulean assemblage (*n* = 1074) with simple core reduction strategies, overlain by an assemblage (*n* = 5565) with organized core reduction strategies [29]. The Victoria West assemblage (*n* = 8604) then overlies the organized core assemblage layer, which is about 2 m thick in Pit 6 (figure 1*c*,*d*). It contains a large number of flakes and flaking debris, constituting the bulk of the assemblage (89.6%; *n* = 7711). Formal tools that are shaped or retouched, including 118 LCTs, compose 5.5% (*n* = 471). The Victoria West cores (*n* = 30) compose 8.2% of the core sample, while simple cores and organized cores together account for 91.8% (*n* = 337). Simple cores are those without any preparation, often showing a relatively short reduction sequence, while organized cores show some concept of volumetric organization in the flaking process, but they lack the pre-planned consistent shaping of the final removal [33,34]. Five stratigraphic layers are recognized according to the sediments. All Victoria West cores were excavated from the coarse alluvial deposits of layer B, which is overlain unconformably by a mantle of younger sands that are largely aeolian in origin [35]. Dating samples collected from the lower part of layer B have provided a preliminary age of approximately 1 Ma, with the Al^26^/Be^10^ method [29], but these dates remain unpublished as further work is needed to confirm them. Therefore, we follow Beaumont and Vogel's [28] age of 0.8–1.1 Ma for the Victoria West at Canteen Kopje, which is based on comparison of the nearby site of Doornlaagte where palaeomagnetism has been used.

Our study sample consists of 30 Victoria West cores in the upper gravel layer. The burial depth of each specimen is shown in electronic supplementary material, table S1 (see electronic supplementary material). Andesite is the predominant raw material used in the assemblage (94.5%), while hornfels is 1.6%, and a variety of minor raw materials make up the remaining 3.9%. Andesite occurs in the nearby koppies and is also the main rock type in the gravels of the ancient Vaal River palaeochannel (figure 1*b*). Victoria West cores are made exclusively in this material, with boulder-sized clasts being commonly used. The availability of raw material was an attraction for the hominids who repeatedly visited the site [31]. The local river and nearby open country resources must also have been very significant for subsistence during Acheulean occupations.

### 2.2. Methods

**Figure 2.**
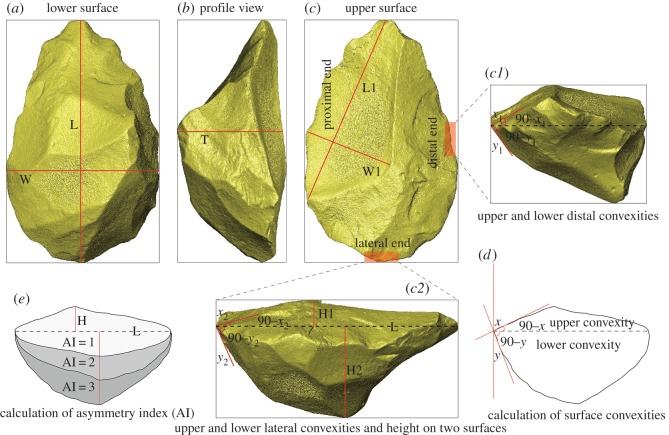
Methodology for the Victoria West core analysis with the aid of three-dimensional scanning. (*a*–*c*) Show the core orientations and size measurements for both the entire core and the preferential flake. (*c1*, *c2*, *d*) Show the calculation of distal and lateral convexities for the upper and lower surfaces of the core. (*c2*) Also shows the measurements of height on the upper core surface (H1) and lower core surface (H2). (*e*) Shows a model with the asymmetry index (AI) at 1, 2 and 3.

All Victoria West cores were scanned using the computed tomography (CT) scanner housed in the Evolutionary Studies Institute of the University of the Witwatersrand, allowing us to scan very large specimens. Three-dimensional models of cores saved as STL files were then imported into the Avizo Fire 3D imaging software program for accurate acquisition of the analytic data. The following core orientation definitions are applicable: the upper face of a Victoria West core is defined as the shallower surface containing the final preferential removal, while the opposite face is defined as the deeper lower surface of the core, used to prepare the upper surface; the convergent tip is viewed most easily from the upper surface of the core, at the end of the long axis, where the two core edges converge (figure 2*a*–*c*).

To demonstrate the hierarchical nature of any Victoria West core, we calculate the asymmetry index (AI) between these upper and lower surfaces. The index is calculated by dividing the upper surface height (H1) by the lower surface height (H2). Height is determined as the perpendicular distance from the highest points of each of the two surfaces to the core maximal length line (figure 2*c2*,*d*). Accordingly, a ratio close to 1 means that the two surfaces are more symmetrical, whereas a larger ratio indicates a higher degree of asymmetry (see figure 2*d* for a model with AI values of 1, 2 and 3).

The lateral and distal convexities are also important features of the Victoria West cores. The convexity is expressed by the angles measured in the Avizo Program (figure 2*c1*,*c2*,*e*). The orientation of lateral and distal sides of a core is arranged according to the location of the striking platform for the final large removal. In particular, the side with the striking platform here is defined as the proximal end of the core, while the distal end is the long side opposite the striking platform. The lateral end here is therefore defined as the side between the proximal and distal ends (figure 2*c*,*c1*,*c2*). Only one lateral convexity can be measured due to removal of the convergent tips of the cores in most cases, which originally served as the other lateral end of the cores.

The size of the Victoria West cores and the preferential removals are measured by importing the three-dimensional models into the ImageJ software program (figure 2*a*–*c*). For capturing core shape information, a geometric morphometric analysis is conducted by using the Thin Plate Spline (TPS) software program [36,37]. Specifically, 60 equidistantly distributed semi-landmarks, along the outlines, are recorded on each core. This is then followed by a generalized Procrustes analysis, which helps to exclude non-shape information through minimizing the Procrustes distance among the corresponding semi-landmarks [38]. Finally, relative warp analysis, which is equivalent to a principal component analysis, is conducted to examine the primary patterns of shape variation [39]. TPS-grids are also used to illustrate the variations along each principal component.

In general, CT scanning of the Victoria West cores in this study provides an effective three-dimensional approach to quantitatively calculate the asymmetrical degree and the differential convexities of the two surfaces of cores. In addition, a geometric morphometric analysis of the shape of Victoria West cores can be easily and accurately achieved by using the scanned models of those cores.

## 3. Results

### 3.1. The hierarchical asymmetrical surfaces

**Figure 3.**
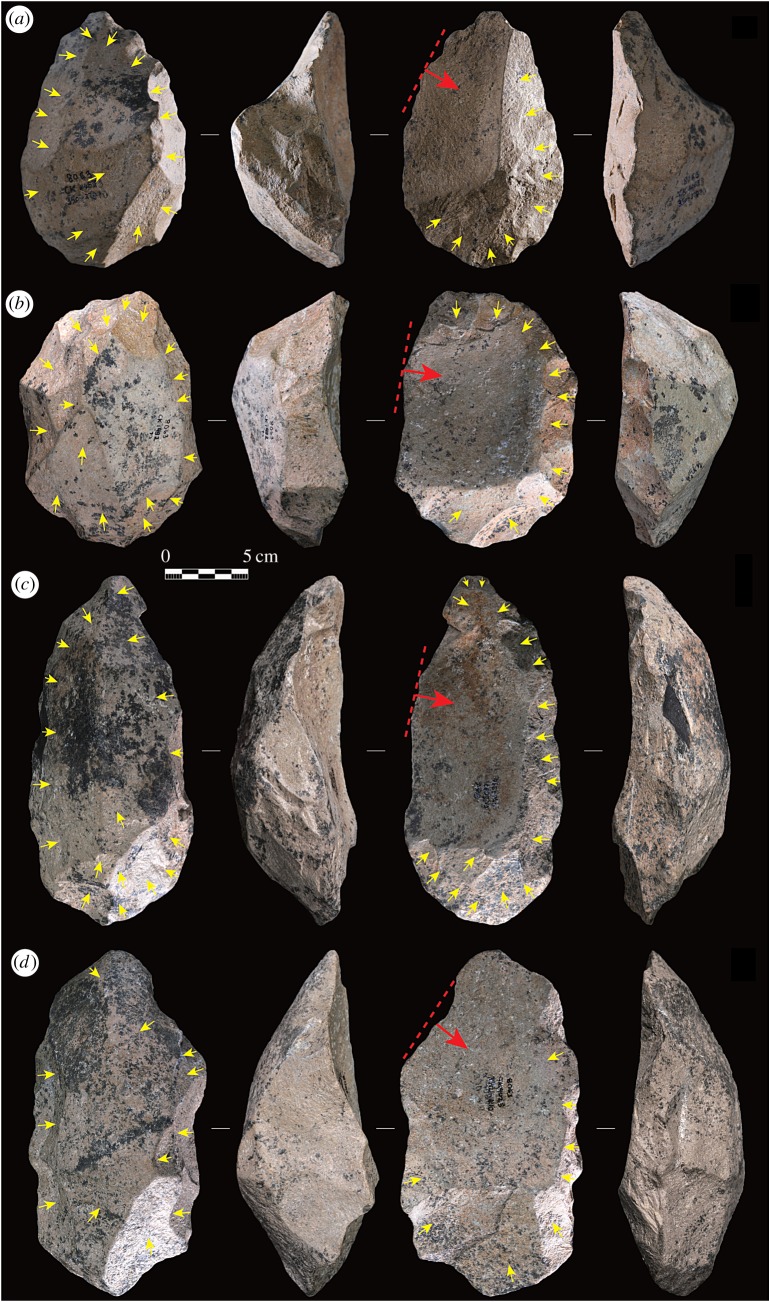
Victoria West cores showing cleaver-like preferential removals on the upper flaking surfaces (the third row). The yellow arrows show the preparation of the two surfaces, the red arrows show the preferential flake removal directions, and the red dashed lines provide an estimate of the striking platform outlines. (*a*–*d*) Correspond to the specimens CK4453, CK1492, CK8090 and CK9025 respectively (see electronic supplementary material, table S1 for raw data).

**Figure 4.**
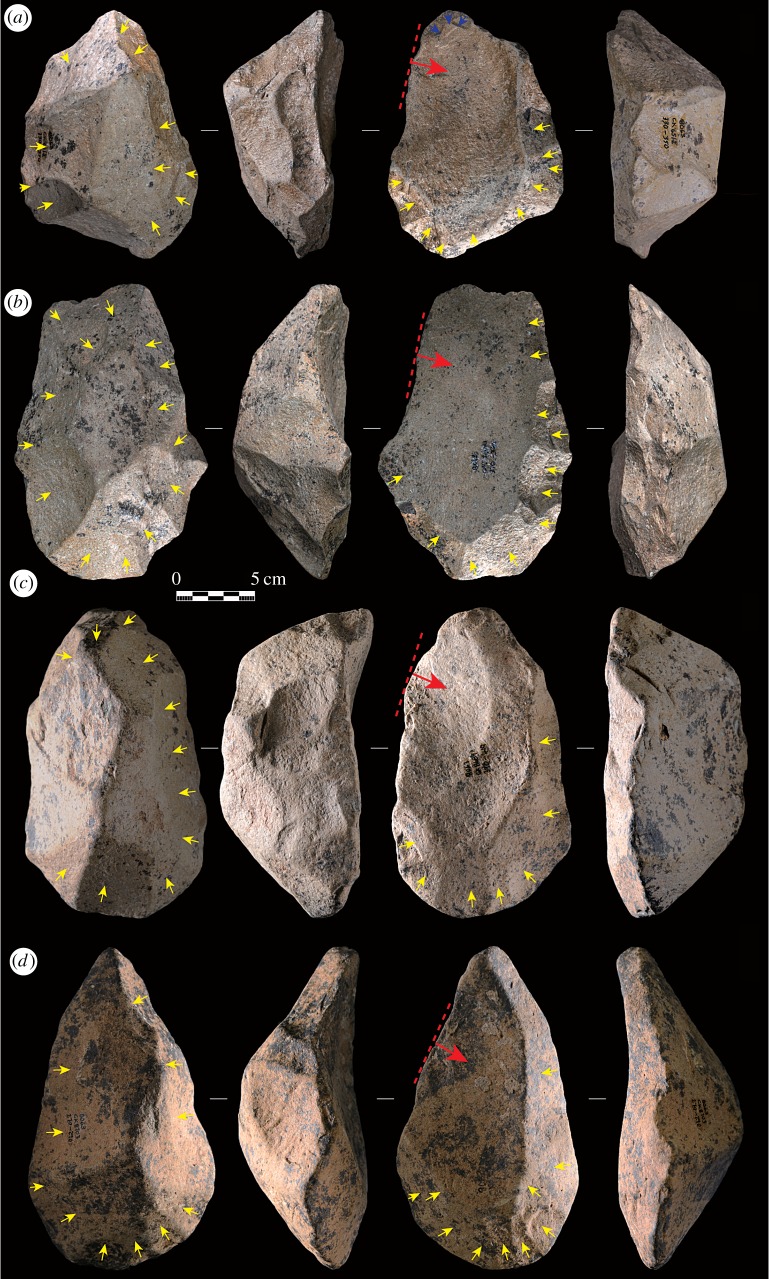
Victoria West cores showing preferential removals with convergent shapes (the third row). The yellow arrows show the preparation of the two surfaces, the red arrows show the preferential flake removal directions, the red dashed lines provide an estimate of the striking platform outlines, and the blue arrows show scars overlapping the preferential removal. (*a*–*d*) Correspond to the specimens CK4512, CK7400, CK8689 and CK8703 respectively (see electronic supplementary material, table S1 for raw data).

According to the Levallois Volumetric Concept proposed by Boëda [40,41], the volume of a prepared core is shaped in terms of two intersecting asymmetrical surfaces. These two surfaces are hierarchically organized and cannot be reversed during the knapping process. The upper surface of the core is regarded as the flaking surface, which serves for detaching the predetermined flake blank. The lower surface serves as a striking platform for preparing the upper surface, and its volume is always larger than the upper flaking surface. This spatially organized strategy of core reduction is found in the Victoria West cores (see figures 3 and 4 for examples). The second row of figures 3 and 4 clearly shows that the upper surfaces are very flat compared with the lower surfaces, which are more arched or even pyramidal in some cases (see figure 3*a* for an example). Quantitatively, the mean asymmetry index of 3.0 (s.d. = 1.2) indicates that there is dramatic asymmetry between the two surfaces. The non-parametric Mann–Whitney *U* test also shows that there is a statistically significant difference (*p* < 0.0001) in height between the upper and lower surfaces (see electronic supplementary material, table S1 for the raw data). The result confirms the hierarchical relationship of the upper and lower surfaces and thus demonstrates the early emergence of a complex spatial cognitive concept. This concept is well beyond the organized core reduction strategies dating to 1.3 Ma at the nearby site of Rietputs 15 [34].

### 3.2. Lateral and distal convexities and surface preparation

Some have discussed the importance of maintaining the lateral and distal convexities of a core during reduction, which can effectively guide the shock wave during percussion and give rise to a predetermined flake removal [40–42]. This is another important component of the Levallois Volumetric Concept and the Victoria West cores present this same pattern. This is investigated by measuring the lateral and distal core angles (figure 2*c1*,*c2*,*d*). The mean lateral angle on the upper surface is 20.4° (s.d. = 5.7), and the mean distal angle on this surface is 28.1° (s.d. = 6.0). The mean angle values are 47.9° (s.d. = 8.5) and 48.8° (s.d. = 5.7) for the lower lateral and distal ends, respectively. Compared to the upper surfaces, the angles of lower surfaces are obviously less acute. This demonstrates the different preparation strategies that apply to the two distinct surface volumes. Such a volumetric concept, with differing convexities between the two surfaces, is consistent with MSA Levallois technology.

Centripetal knapping of both the upper and lower surfaces, to achieve the appropriate morphological characteristics, is another preparative procedure comparable with the MSA Levallois. The yellow arrows marked in the first and third rows of figures 3 and 4 clearly show this pattern. Extensive knapping occurs on both surfaces, with a mean scar number for upper surfaces at 9.5 (s.d. = 3.9) and for lower surfaces at 14.8 (s.d. = 6.3). Considering that the final large removals probably take off some scars from the upper core surface, the mean scar count on this surface can be somewhat higher than what the data indicate here.

### 3.3. Method for detaching the predetermined flakes

Levallois methods for the detachment of predetermined flakes are diverse, including preferential, recurrent unidirectional, recurrent bidirectional and recurrent centripetal [40,41,43–45]. Compared with this considerable variety of exploitation methods, the Victoria West cores, however, show an exclusive method of preferential flaking. Only one large flake is detached, nearly always, from the upper left side of the upper core surface. Such a pattern occurs repeatedly in all successfully flaked cores (figures 3 and 4). As a consequence of this strategy, the flakes that are successfully removed have technological length measurements that are shorter than their widths. The negative flake impressions on the upper core surface clearly show these dimensions. This kind of flake is defined as ‘side-struck’, following Sharon's study of Acheulean large flake blanks [46,47]. In addition, the negative flake impressions indicate that the direction of flaking, for the preferential flakes, originates from a perpendicular or only slightly oblique angle in relation to the striking platform (see the red arrows and red dashed lines in figures 3 and 4). Platform preparation, usually through faceting, is hard to determine due to the removal of platforms from cores during the flaking process. However, according to the parallel, or sub-parallel, nature of the observed negative flake impressions in relation to the planes of intersection of the two surfaces, we can reasonably infer that the platform angles should be steeply faceted to acquire the large parallel removals. Freehand hard hammer percussion is used for flake detachment, as shown by the robust negative bulb scars preserved on some cores (figures 3 and 4).

Failed or undesired flake detachments also occurred in several cores, although in a relatively small number. There are three specimens with overshot preferential removals (electronic supplementary material, figure S1*a*–*c*), yet these flake blanks would nevertheless have still had the potential to make LCTs, regardless of their non-uniform shape. Four specimens in the core sample show small preferential flake detachments, and perhaps these were too small to serve as LCT blanks (see electronic supplementary material, figures S1*d* and S2*a*–*c*). However, the morphology of these cores provides robust evidence that confirms the intentional hierarchical preparation of the two surfaces. Lastly, in one core the striking direction appears to have been moved intentionally to originate from the lower left side of the upper surface, most likely due to the unexpected internal joints in the raw material (see the yellow dotted circle in electronic supplementary material, figure S2*d*).

### 3.4. Size and shape characteristics

The size of the Victoria West cores is variable: length ranges from 87.0 to 282.1 mm (mean = 184.9, s.d. = 38.9), width ranges from 47.6 to 147.2 mm (mean = 107.2, s.d. = 19.3) and thickness ranges from 35.0 to 104.0 mm (mean = 70.7, s.d. = 13.8). Core weight ranges from 161.7 to 4830.0 g (mean = 1573.1, s.d. = 818.3). Core size and weight variability indicates the ability of hominids to adapt to the naturally occurring size of the raw material blanks. With respect to the size of the preferential removals, excluding the four small undesired detachments, these flakes have a length range of 70.6–165.3 (mean = 132.6, s.d. = 25.1) and a width range of 44.5–126.7 (mean = 81.1, s.d. = 17.2). Therefore, size variability in the preferential flakes is also apparent. Because the convergent tips of the cores have been removed during the final strike in some cases, the real length for the entire cores and for the preferential removals should be larger than what the current data indicate. The average length of the preferential flakes in our sample confirms that the assemblage belongs to the ‘Large Flake Acheulean’ tradition, in which the maximal length of flakes is larger than 10 cm [48,49]. In addition, the average size of those large flakes is also consistent with the average size of handaxes (13.8 in length and 7.9 in width) and cleavers (13.3 in length and 7.7 in width) in this assemblage, providing evidence that those flakes are used as blanks to make Acheulean large cutting tools [29].

**Figure 5.**
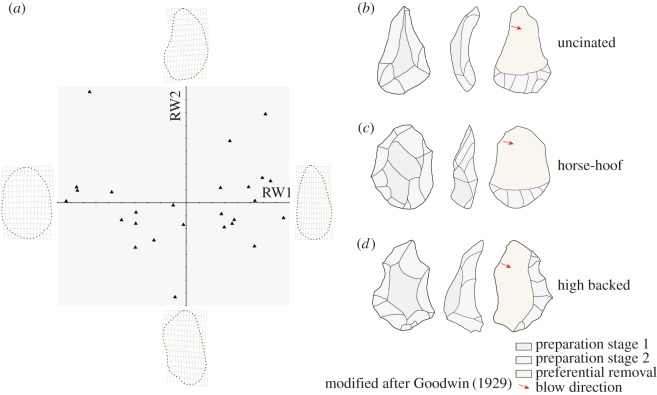
(*a*) Showing relative warp analysis of the Victoria West core shape, while the (*b*–*d*) shows the three types of Victoria West cores, initially identified by Jansen and Goodwin.

In the earliest study of Victoria West cores from surface collections, Jansen [22] and Goodwin [24] identified three morphological types, namely the uncinated (or hoenderbek), horse-hoof and high backed (figure 5*b*–*d*). Through our analysis we also find that there is a consistent shape pattern in the Canteen Kopje Victoria West cores. Specifically, the uncinated shape, which is characterized by a narrow convergent tip and the relatively wide and arched bottom, is dominant throughout the sequence (*n* = 17; see electronic supplementary material, figure S3 for examples from the top of the layer). Such a pattern is reminiscent of the shape characteristic of handaxes. Only one example has an original core shape close to the horse-hoof in our excavated sample (figure 3*b*). In some other examples where the current shape is also similar to the horse-hoof (see figure 4*b* as an example), it is difficult to judge the original shape due to the removal of the ends of the core during blank detachment. Considering the consistency in core shapes, these examples would most likely have been uncinated, originally, prior to final flake detachment. The high-backed type (see figure 4*a* as an example) proposed initially by Jansen [22] and Goodwin [24], in our opinion, should be discarded as both the uncinated and horse-hoof cores can possess a high back, which is highly related to the hierarchical preparation of the two core surfaces.

Relative warp (RW) analysis of core shape supports this observation. Half of the specimens with elongated pointed shapes (i.e. the uncinated shape) plot in the positive-value area of RW1 (45.6% of variance). Another half plot in the negative-value area and is associated with a relatively elongated ovate shape (equal to the horse-hoof type), which we suggest is mainly due to the removal of narrow distal ends for these specimens. The specimens along RW2 (16.6% of variance) have a similar elongated shape with few variations (figure 5*a*).

With regard to the shape of the preferential removals, two main types are identified according to the preserved negative flake impressions on the upper core surfaces. The first is a straight or slightly convex cleaver-like shape (see figure 3 for examples), which we suggest is desirable for making cleavers. The second is a convergent pointed shape (see figure 4 for examples), which is probably used to make handaxes, although we cannot deny the possibility of using the wide oblique edges of these blanks to make cleavers.

## 4. Discussion

### 4.1. Similarities to and differences from the Levallois and implications for the origins of prepared core technology

The relationship between the Victoria West and the Levallois is a significant but highly debated issue. Some scholars support the ancestor-descendant relationship and prefer the term ‘proto-Levallois’ for the Victoria West cores [23–27], while others argue that the emergence of the Victoria West core is a result of convergent technological evolution and the term ‘para-Levallois’ is more appropriate, as argued in Lycett's phylogenetic study [32,50]. However, regardless of which opinion one holds, all researchers recognize that the Victoria West is a highly standardized prepared core reduction strategy (e.g. [51–53]), and our study further confirms this point. Compared with the Levallois Volumetric Concept proposed by Boëda [40,41], similar procedures are evident in the preparation and reduction of Victoria West cores and these include: (i) careful radial knapping of two hierarchically related asymmetrical surfaces; (ii) maintenance of lateral and distal convexities for the detachment of the predetermined preferential flakes; (iii) the sub-parallel angle of the large negative flake impressions in relation to the plane of intersection of the two core surfaces; (iv) the nearly perpendicular orientation of the striking platforms relative to the technological axes of preferential flakes; and (v) freehand hard hammer percussion for the final removals.

In addition to these similarities, unique features presented in the Victoria West cores are also clear and are sufficient to define them as a separate form of prepared core technology. First, these cores are frequently large and shapes are predominantly convergent-pointed, similar to the shape of handaxes. Such a shape is good for detaching large flakes, with preferential long axes. Second, the blow direction of the final removals is consistent and originates from the upper left side of the upper core surface. Third, large side-truck flakes, whose technological length is shorter than the technological width, are the primary end products. Fourth, and different from the Levallois flakes, points or blades used for making small tools (e.g. scrapers), large flakes are detached from the Victoria West cores and may have been used as blanks to make Acheulean LCTs, such as handaxes and cleavers, or they may have been used as knives without further modification.

Therefore, both similarities and differences exist in the Victoria West cores, when compared with the Levallois. The Victoria West industry is clearly Levallois in character and has a deserved place in the development of prepared core technology. The age estimate for the Victoria West industry is the earliest known prepared core technology in the world [28,29], as it exceeds the age of the prepared cores at Gesher Benot Ya'aqov site in Israel at 0.7/0.8 Ma [54,55]. Although other Acheulean sites, such as Tabelbala in Algeria and Chirki in India, are also known for the existence of giant prepared cores, used for detaching blanks for LCTs, the age of these sites has never been determined and they are estimated to belong to the Middle Pleistocene [56–58]. Irrespective of the lack of dating work at most of these sites, we argue that the Victoria West is currently the earliest prepared core technology in the world [28,29].

In comparison with the preceding Oldowan technocomplex, the appearance of LCTs around 1.76 Ma has been suggested as a significant innovation and behavioural change [6,7,59]. The Victoria West, when viewed from this perspective then, undoubtedly represents another important innovation and technological advancement during the Early Acheulean, demonstrating the origins of prepared core technology in Africa. It is also significant that the Victoria West industry shows a degree of continuity with the earlier nearby site of Rietputs 15 at 1.3 Ma, where an organized core reduction strategy exists [34]. This suggests a regional development over space and time. The high degree of planning exhibited by the Canteen Kopje Victoria West cores provides robust material evidence for our understanding of the cognitive and social evolution of early hominids, as discussed below.

### 4.2. Implications for hominid cognitive evolution

Understanding the cognitive evolution of hominids is a highly debated topic, not only for Palaeolithic archaeologists, but also for evolutionary anthropologists and psychologists. To date, several influential models and/or hypotheses have been proposed to account for stages in our evolutionary development and these include: the Working Memory Model, the Identity Model, the Social Brain Hypothesis, and the Visual Display Hypothesis [60–66]. However, the cognition of hominids in the evolutionary stage of pre-modern humans is still fairly speculative, when compared with the intense studies of the emergence of modern behaviours and cognitive ability, whose archaeological records are relatively abundant and diverse. As the earliest prepared core technology, the Victoria West cores will provide new evidence for a meaningful assessment of the cognitive evolution of hominids in such an early period.

The sample in this study contains 30 excavated Victoria West cores from a burial depth of approximately 2 m [29]. Compared with the relatively large populations that may have contributed to this sample, the number of individuals motivated to produce this technology is probably small, although others may also have been capable of such thought. This can be a possible reason for the small number of Victoria West cores. Regardless of the number, the systematic and standardized preparation process evident in the Victoria West cores suggests that a mental template existed in the minds of hominids. We believe that this mental template reflects sophisticated abstract thinking by individuals and a teaching-learning mechanism within a social context. From a *chaîne opératoire* perspective, such a template is not isolated, but it serves as an inter-related link between the initial selection of raw materials up until the final stages of LCT production. The occurrence of deep planning, for distinct core production stages, directly demonstrates this increase in cognition and complexity. In addition, the enhanced cognitive demands for making Victoria West cores may have a close relationship with the relatively long-term working memory evident in the development of the brain's prefrontal cortex [66]. Such a process of encephalization is suggested to be driven by social complexity, which is partly expressed in the growing size of social groups and the closer cohesion of individuals [14,60]. Those changes to the social life of the Canteen Kopje hominids, as a consequence, probably ensured the inheritance of Victoria West technology through generations in this specific area of interior South Africa.

The Victoria West cores represent an innovative and advanced technological expression. Nevertheless, compared with MSA Levallois prepared cores, some disparities can also be revealed. Cores produced by the Levallois are well documented by their diversification, both in their patterns of flaking and the different degrees of core preparation. In addition to preferential flaking, recurrent unidirectional, recurrent bidirectional and centripetal flaking are also identified as important forms of Levallois core reduction [43–45]. Simple prepared cores that lack one or more criteria as defined by Boëda [40,41] are also present in these industries. The use of natural raw material convexities to detach predetermined flakes is a good example [45]. By contrast, the Victoria West cores show a mainly monotonous nature. Although the size and shape of both the cores and the final preferential removals are varied, the application of technological procedures is considerably uniform: andesite is used exclusively; only one large preferential flake is struck, and from a consistent direction; the overall shape of the Victoria West cores is predominately elongated convergent. All of these features imply a lack of technological flexibility when compared with MSA Levallois technology.

## 5. Conclusion

Nearly a century has passed since the first recognition of Victoria West technology. However, the significance of this technology, especially its role in understanding the origins of prepared core technology and the cognitive evolution of early hominids, is still not fully appreciated and understood. Our study at Canteen Kopje shows that the Victoria West cores, on the one hand, bear similarities to the Volumetric Concept of the Levallois, while on the other hand, this technology contains features that are sufficient to define the Victoria West as a unique prepared core technology. The antiquity of these Victoria West cores indicates that the origins of prepared core technology have a deep root in the Early Acheulean, much older than previously known. The Victoria West represents the emergence of complex cognitive abilities, but this kind of ability is still relatively less sophisticated than the later, more diversified Levallois technology. This implies an evolutionary trajectory of human cognition from the Victoria West to Levallois, and finally to the emergence of modern humans' thinking, which we suggest is hard-wired in the cognition of these early hominids.

## Supplementary Material

Victoria West cores

## Supplementary Material

Victoria West cores

## Supplementary Material

Victoria West cores

## Supplementary Material

Raw data of Victoria West cores

## Data accessibility

The Victoria West cores studied in this paper are stored at the Archaeology Department of the University of the Witwatersrand, Johannesburg.
