# Supplementary figures and images for: The Victoria West: earliest prepared core technology in the Acheulean at Canteen Kopje and implications for the cognitive evolution of early hominids
Source: R Soc Open Sci. 2017 Jun 28;4(6):170288. doi: 10.1098/rsos.170288 (PMC5493924; doi:10.1098/rsos.170288)

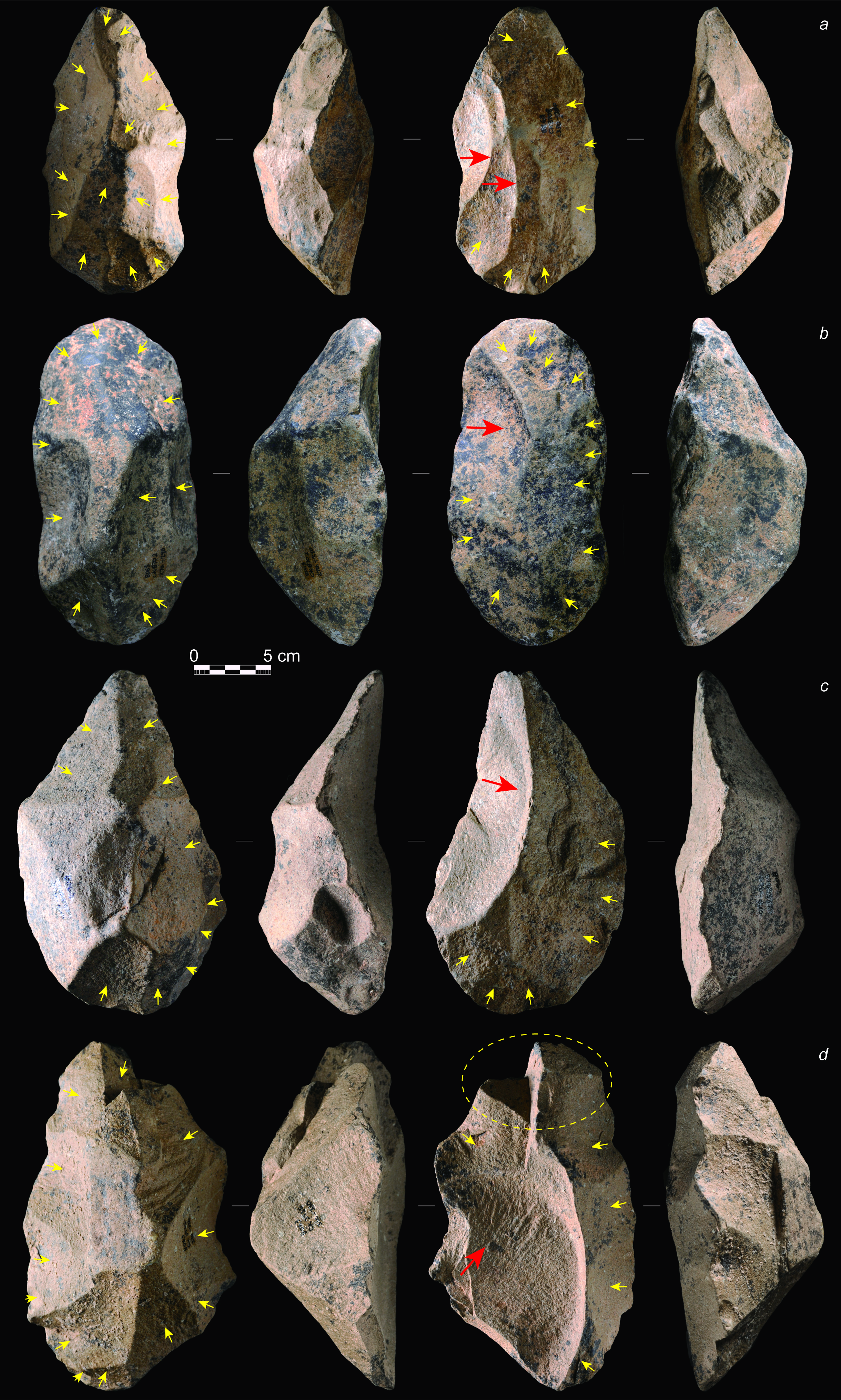

Supplement: Victoria West cores [file rsos170288supp1.jpg]

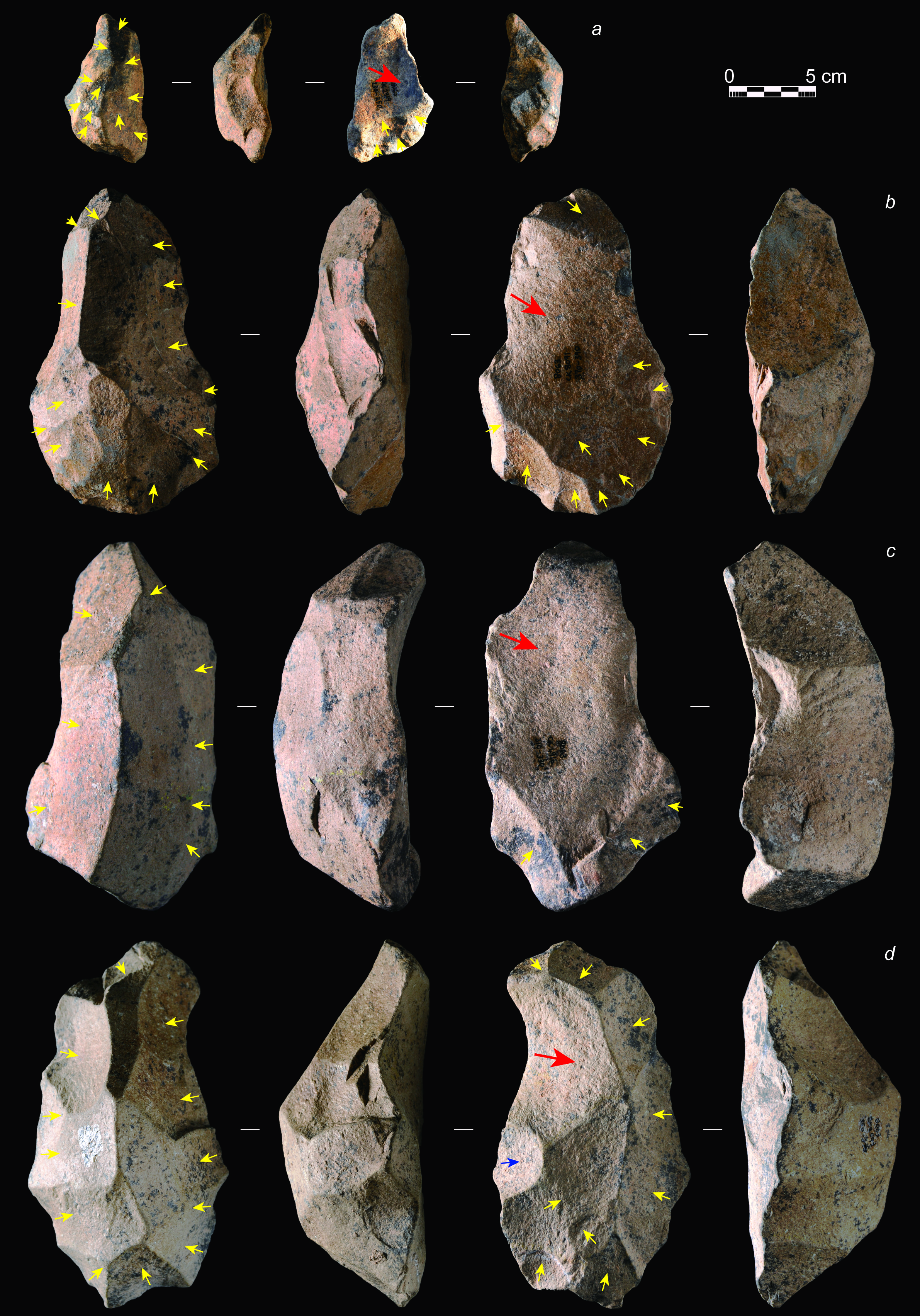

Supplement: Victoria West cores [file rsos170288supp2.jpg]

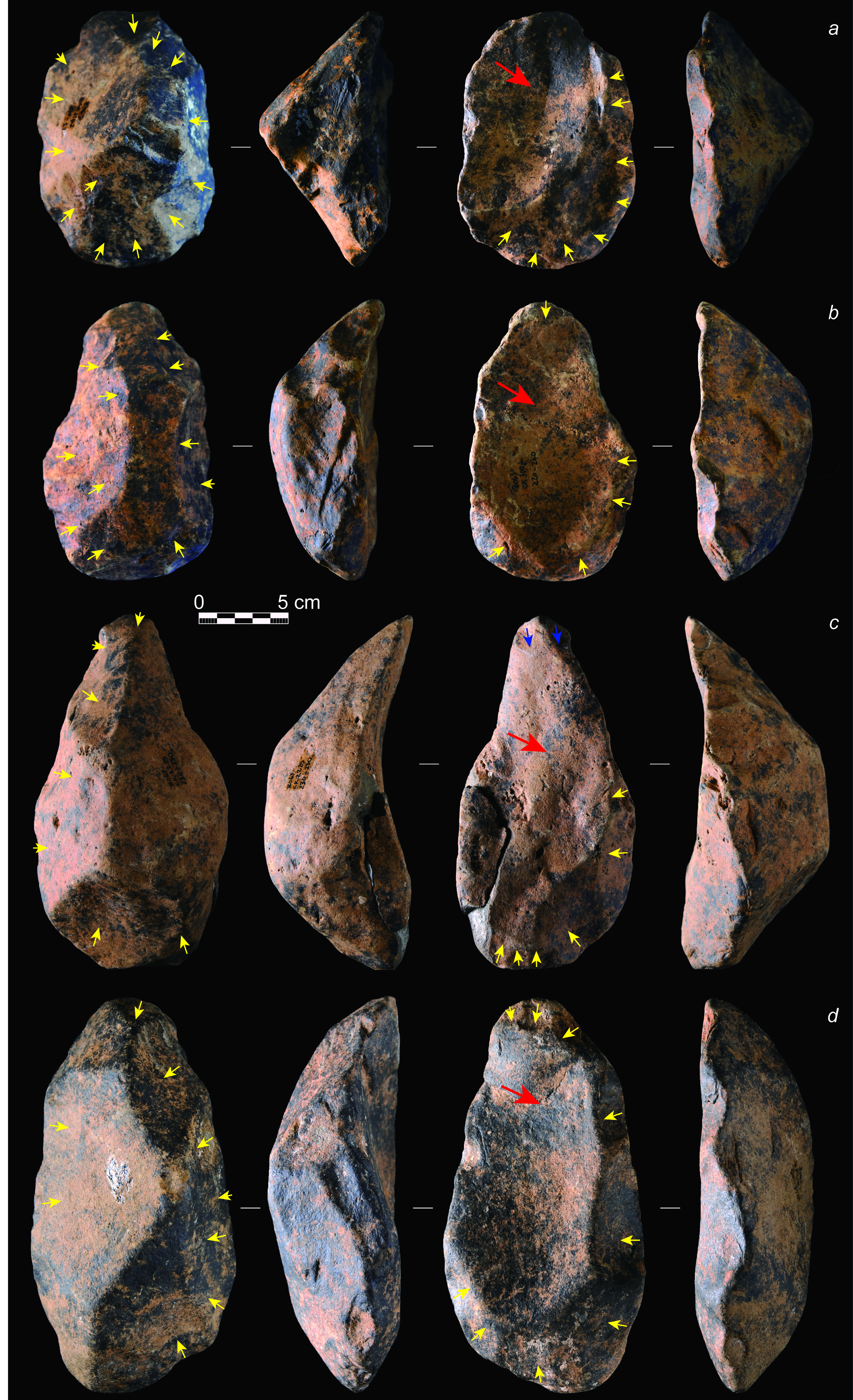

Supplement: Victoria West cores [file rsos170288supp3.jpg]
